# Redescription of *Gnorimosphaeroma oregonense* (Dana, 1853) (Crustacea, Isopoda, Sphaeromatidae), designation of neotype, and 16S-rDNA molecular phylogeny of the north-eastern Pacific species

**DOI:** 10.3897/zookeys.1037.63017

**Published:** 2021-05-13

**Authors:** Regina Wetzer, Adam Wall, Niel L. Bruce

**Affiliations:** 1 Research and Collections Branch, Natural History Museum of Los Angeles County, 900 Exposition Boulevard, Los Angeles, California 90007, USA Natural History Museum of Los Angeles County Los Angeles United States of America; 2 Queensland Museum, Brisbane, Australia Queensland Museum Brisbane Australia; 3 North-West University, Water Research Group, Unit for Environmental Sciences and Management, Private Bag C6001, Potchefstroom 2520, South Africa North-West University Potchefstroom South Africa

**Keywords:** Brackish, California, East Pacific, freshwater, intertidal, San Francisco Bay, Tomales Bay, Washington

## Abstract

*Gnorimosphaeroma oregonense* (Dana, 1852) is revised, a male neotype is designated, photographed, and illustrated; the species occurs from Vancouver British Columbia to the central California coast. 16S-rDNA sequences (~650 bp) for all available ethanol preserved species of *Gnorimosphaeroma* were used to hypothesize their relationships. Our analyses revealed a sister taxon relationship between the fully marine *G. oregonense* and the brackish to freshwater species, *G. noblei*. The oyster associated and introduced *G. rayi* is sister to a previously not recognized or identified, but genetically distinct, *Gnorimosphaeroma* sp. collected at two sites in San Francisco Bay. *Gnorimosphaeroma* sp. is probably also a western Pacific species based on its genetic relationship to *G. rayi*. Photographic comparisons are offered for *G. oregonense* (marine), *G. noblei* (freshwater), *G. rayi* (introduced), *G.* sp. (presumably introduced), and *G. insulare* (San Nicolas Island). Records of the holdings at the Los Angeles County Museum of Natural History are summarized. Without material available north of Vancouver through Alaska, the range of *G. oregonense* could not be genetically verified. This review includes a diagnosis and description of the genus *Gnorimosphaeroma* Menzies, 1954.

## Introduction

In the temperate region of the East Pacific, the sphaeromatid isopod fauna is limited to shallow coastal waters and is represented by eight genera (*Dynoides* Barnard, 1914, *Dynamenella* Hansen, 1905, *Exosphaeroma* Stebbing, 1900, *Gnorimosphaeroma* Menzies, 1954, *Paracerceis* Hansen, 1905, *Paradella* Harrison & Holdich, 1982, *Pseudosphaeroma* Chilton, 1909, and *Sphaeroma* Bosc, 1801). The genus *Gnorimosphaeroma* Menzies, 1954 was erected for three species and a subspecies. *Gnorimosphaeroma oregonense* (Dana, 1853) was designated as the type species. Menzies (1954) further distinguished *G. oregonense oregonense* from *G. oregonense lutea*, a new subspecies, from the west coast of North America from, “very brackish to almost freshwater”. In his 1954 paper, Menzies included *G. insulare* (Van Name, 1940) collected from freshwater on San Nicolas Island (California Channel Islands); described *G. noblei* from marine waters in Tomales Bay, California; *G. chinensis* (Tattersall, 1921) from freshwater in Shanghai, China; *G. ovata* (Gurjanova, 1933) from marine environments off Japan; and recognized a *Gnorimosphaeroma* sp. an undescribed species from Japanese seashores. Today 26 species are accepted by the World Register of Marine Species (Boyko et al. 2008). The genus is restricted to the northern Pacific from Japan and China to Alaska and California. *Gnorimosphaeroma* is unusual among sphaeromatids as it contains marine as well as fresh- and brackish-water species (Menzies 1954).

Dana’s (1853) redescription and accompanying figures for the type species of *Gnorimosphaeroma oregonense* are inadequate to distinguish the species. All of Dana’s isopod specimens were lost when the sloop, the USS ‘Peacock’, sank at the mouth of the Columbia River on July 18, 1841 (Hanable 2003; Bruce 2009: 211) and the type material is unequivocally lost. Menzies (1954) erected *Gnorimosphaeroma* for Dana’s species, but did not designate a neotype for *Gnorimosphaeroma oregonense* (Dana, 1853), providing only a redrawn figure of a portion of the pleotelson. Menzies’ attribution of the species range from the Bering Islands, Alaska to and including San Francisco Bay, further precludes precise inclusion of what constitutes the taxon, as this large geographical range likely includes more than one species.

Here we review specimens attributed to *Gnorimosphaeroma* from Vancouver, Canada and the state of Washington to Santa Barbara, California and to the southern California offshore island San Nicolas, from fully marine to freshwater habitats. We provide a 16S-rDNA phylogenetic hypothesis of the relationships for all of the material at hand, designate a replacement for the lost *Gnorimosphaeroma oregonense* type specimen, and redescribe the species. Furthermore, we provide comparative photographs of *G. oregonense*, *G. noblei*, *G. rayi*, *G. insulare*, and *Gnorimosphaeroma* sp.

## Materials and methods

The redescription of *Gnorimosphaeroma oregonense* is based on the male neotype (here designated) and additional material as described below. Specimens examined have LACM numbers preceded by RW which are field station numbers. Collections so labelled are readily retrieved from the LACM collections as are those denoted as DISCO. Setal terminology broadly follows Watling (1989).

Examined specimens were obtained from 49.294°N (British Columbia) to ~33.262°N (California). *Gnorimosphaeroma* material held in the LACM collections and available for morphological study is presented in Table 1. Some material is available for both morphological and genetic examination (Table 2). We sequenced all material preserved and useful for molecular analysis resulting in Figures 10 and 11 and photographed representatives of these four species including a fifth species discovered during the genetic analyses (Figs 12–15).

**Table 1.** Museum collections examined morphologically and not included in the molecular analyses. Taxa are grouped by species and sorted by latitude. Label data and associated notes are transcribed here. Note that in some instances latitude and longitude are approximate and are indicated as “~”. Although we attempted to extract and amplify DNA, some were unsuccessful.

Species	Specimen label
*Gnorimosphaeroma insulare*	California, Ventura County, San Nicolas Island, ~33.262°N; ~119.502°W, fresh water pond with pulmonate mollusk, *Physa virgata* Gould, 1938, Types at AMNH 8092, one syntype at LACM CR 1938-270.1, Coll. T.A.D. Cockerell, Collection ID: RW17.013
*Gnorimosphaeroma noblei*	California, Humbolt County, Humboldt Bay, *Salicornia* flats, 3/4 mi N of Samoa, ~40.858°N; ~124.153°W, mud banks, preserved in 70% ethanol, 29 Apr 1972, I72-30, MBPC 6774, Coll. R. Talmadge & E. Iverson, Collection ID: RW17.044
	California, Mendocino/Sonoma County, 100 yds. up from mouth of Russian River, ~38.437°N; ~123.11°W, preserved in 75% ethanol, 19 Aug 1971, EI-1969, Coll. J. Carlton, Collection ID: RW17.047
	California, Sacramento County, central San Joachin Delta (freshwater), ~38.33°N; ~121.3°W, collected before May 2003, fixed in formalin, preserved in 70% ethanol, Coll. Wayne Fields, Collection ID: RW03.218
	California, Sacramento County, central San Joachin Delta (freshwater), ~38.33°N; ~121.3°W, collected before May 2003, fixed in formalin, preserved in 70% ethanol, Coll. Wayne Fields, Collection ID: RW03.217
	California, Marin County, Tomales Bay, at the Marconi Marina, ~38.143°N; ~122.879°W, under rocks with *Armadilloniscus* at high tide line, preserved in 75% ethanol, 21 Feb 1972, C72-19, SDNHM A.0030, NHM36, MBPC 6783, Coll. Ernie Iverson and J. Carlton, Collection ID: RW14.069
	California, San Joaquin County, Delta-Mendota Canal, mile post 11.35, ~37.991°N; ~121.263°W, freshwater; Isopods very abundant in clusters and as individuals all along surface (concrete wall) and in mass congregation in darkened cracks/crevices. These scooped up in one small hand-full. Canal running at high-water and fully operating: water at high velocities (12?-13? mph surface velocity). Some isopods observed crawling slowly against this current. Some of the larger specimens collected also by hand elsewhere in the same area along concrete wall, preserved in 75% ethanol, 6 Jun 1972, Coll. J. Chapman & E. Iverson, Collection ID: RW17.046
	California, Marin County, creek at Bolinas Lagoon immediately north of Audubon Canyon Ranch where creek goes under road, 37.925°N; 122.676°W, under rocks, preserved in 75% ethanol, 21 Feb 1972, C72-14, Coll. E. Iverson & J. Carlton, Collection ID: RW17.052
	California, Marin County, creek at Bolinas Lagoon immediately north of Audubon Canyon Ranch, 37.924°N; 122.675°W, brackish creek mouth, preserved in 75% ethanol, 21 Feb 1972, C72-13, Coll. E. Iverson & J. Carlton, Collection ID: RW17.050
	California, Santa Cruz County, San Lorenzo River, City of Santa Cruz, 200–250 m downstream of Laurel Street, 1.5 mi. above ocean, 36.969°N; 122.022°W, fixed in formalin and preserved in 75% ethanol, 22 May 2004, CCS2004-18, Coll. Camm Swift and Steve Howard, Collection ID: RW04.268
	California, Santa Cruz County, San Lorenzo River, ~36.58°N; ~122.03°W, collected before May 2003, fixed in formalin, preserved in 70% ethanol, Coll. Christopher Rogers, rcvd. from Wayne Fields, Collection ID: RW03.216
	California, San Luis Obispo County, Diablo Cove, ~35.211°N; ~120.86°W, preserved 75% ethanol, 19 Apr 1976, Coll. D. W. Behrens, Collection ID: RW17.037
	California, Santa Barbara County, El Capitan State Beach in kelp debris at mouth of Cañada del Capitan, 34.458°N; 120.022°W, preserved in 75% ethanol, 28 Dec 1971, I71-90, Coll. E. Iverson, Collection ID: RW17.051
	California, Santa Barbara County, San Miguel Island, ~34.101°N; ~120.379°W, preserved in 70% ethanol, 11 Oct 1978, Coll. Eric Hochberg, Collection ID: RW17.030
	California, Ballona Creek Estuary, 33.971°N; 118.439°W, Van Veen, 1.5 m, fixed in 10% formalin, preserved in 70% ethanol, 16 Sep 2003, MBPC 10271, Bight ‘03, Sta. 4053, Coll. Aquatic Bioassay and Consulting Laboratories, Inc., Collection ID: RW17.027
	California, Dominguez Channel, 33.802°N; 118.228°W, VanVeen, 4 m, fixed in 10% formalin, preserved in 70% ethanol, 17 Sep 2003, MBPC 10592, Bight ‘03, Sta. 5108, Coll. Kinnetic Laboratories, Inc, Collection ID: RW17.028
*Gnorimosphaeroma oregonense*	Washington, San Juan County, Friday Harbor, Ocean Labs, ~48.546°N; ~22.013°W, marine, night light, 27 Aug 1949, Coll. J.L. Mohr, Collection ID: RW17.039
	Washington, San Juan County, Puget Sound, Seattle Puget Sound Naval Supply Depot, Smith Cove, 47.631°N; 122.386°W, under rocks in sand. LT2, preserved in 75% ethanol, 11 Aug 1973, I73-17, Coll. E. Iverson, Collection ID: RW17.045
	Washington, Grays Harbor County, Grays Harbor, Westport floats, 46.9°N; 124.094°W, on floats among fouling organisms, fixed in isopropyl, preserved in 75% ethanol, 22 Mar 1976, Coll. J. T. Carlton & D. A. Fishlyn, Collection ID: RW17.038
	Oregon, Lincoln County, Cape Perpetua, Strawberry Hill, 44.254°N; 124.112°W, under seaweed at high tide mark, fixed and preserved in 70% ethanol, 9 Jul 1971, rcvd. from Robert Hessler, MBPC 13410, Coll. Fred Schram, Collection ID: RW17.041
*Gnorimosphaeroma oregonense*	Oregon, Coos County, Squaw Island, off Cape Argo Light, 43.339°N; 124.376°W, intertidal, -1.6 ft. tide, rocky reef, some loose rocks kelp covered, preserved in 95% ethanol, 27 Jul 1942, Sta. 1488-42, LACM 42-46.5, Coll. R/V Velero, Collection ID: RW17.033
	California, San Francisco County, San Francisco Bay, Aquatic Park, west of Scout Hut, ~37.8°N; ~122.362°W, under rocks, fixed and preserved in 75% ethanol, 17 Nov 1971, Coll. E. Iverson & J. Carlton, Collection ID: RW17.032

Specimens for SEM were prepared as described in Wall et al. (2015). Drawings were made with the aid of a camera lucida and illustrations were electronically “inked” with Affinity Designer, Serif Labs. Appendages were illustrated by dissecting off the appendage and placing them in glycerol on a depression slide and then imaged using a Nikon Labophot-2 compound scope. Specimens were measured with a micrometer. The lengths given in the “Material examined” are of the largest specimen of each species and sex. Not all specimens were measured. If a length is provided and multiple specimens were present in a lot, the length refers to the largest specimen.

Molecular data was generated according to the protocols described in Wetzer et al. (2013). Voucher specimens are held in the LACMCrustacea Collections. Sequences have been published in GenBank and are summarized in Table 2. Complete metadata is provided in Table 2 for specimens used in the molecular analysis. Our numbering scheme readily allows identification to a specific specimen. Table 1 summarizes specimens examined for morphology. The lot from which the neotype was selected is deposited in the LACMCrustacea Collections. Nexus data will be added to Open Tree of Life upon publication. Wetzer (2015) describes isopod collecting and preservation methods. 16S-rDNA Palumbi et al. (1991) universal 16Sar and 16Sbr primers were used for the 16S-rRNA fragment (~650 bp). Tissue extraction, amplification, sequence editing, sequence assembly as well as alignment protocols are detailed in Wetzer et al. (2013, 2018). The online MAFFT (Multiple Alignment Program for amino acid or nucleotide sequences, Katoh et al. (2002, 2005) alignment tool was used to create separate datasets using LINS, EINS, or GINS alignment protocols. RAxML and MrBayes analyses were performed on CIPRES (Miller et al. 2010).

**Table 2.** Sequences used in the 16S-rDNA analyses are associated with their taxon names in alphabetical order and GenBank accession number. The molecular identification number identifies the specimen on the phylogenetic tree. In several instances multiple individuals were extracted and sequenced from the specimen lot. An asterisk denotes the lot from which neotype was selected.

Species	GenBank No.	Mol. Id.	Specimen label
*Gnorimosphaeroma* sp.	MH427743	2550	California, San Mateo County, Redwood Shores, 631 Marlin Court, ~37.535°N; ~122.249°W, from floating styrofoam boat dock, amongst bases of *Ciona*, salinity 24 ppt, fixed and preserved in 95% ethanol, 9 Nov 2002, Coll. R. Wetzer, N. D. Pentcheff, C. Wetzer, Collection ID: RW02.060
	MH427746	2551	
	MH427744	2550	
	MH427750	3124	
	MH427749	3122	California, Alameda County, San Francisco Bay, off Doolittle Road near Oakland Airport, 37.079°N; 122.224°W, high intertidal, salinity 30 ppt, fixed and preserved in 95% ethanol MBPC: Fixed and preserved in 95% ethanol, 5 Jun 2002, Coll. R. Wetzer and S. Boyce, Collection ID: RW02.030
	MH427747	3120	California, Alameda County, San Francisco Bay, off Doolittle Road near Oakland Airport, 37.731°N; 122.21°W, from high intertidal under rocks, isopods found under rocks most commonly without grapsid crabs – upper intertidal occurring with *Ligia*, salinity 30 ppt, fixed in 95%, preserved in 95% ethanol, 5 Jun 2002, Coll. R. Wetzer, T. Haney, and S. Boyce, Collection ID: RW02.028
	MH427748	3121	
*Gnorimosphaeroma noblei*	MH427755	2546	California, Santa Barbara County, lagoon at mouth of Refugio Creek, Refugio Creek State Park, 14–15 km E. of Gaviota, salinity 0°/°°, ~34.465°N; ~120.069°W, probably fixed in 95%, preserved in 70% ethanol, 22 Oct 1999, Coll. Camm Swift and Todd Haney, Collection ID: RW00.017
	MH427770	3113	
	MH427771	3114	
	KU248168	1541	California, Marin County, Tomales Bay, head of bay near channel (man-made) adjacent to Hwy. 1, 38.091°N; 122.825°W, from under algae and barnacle covered rocks, salinity 20 ppt, fixed and preserved in 95% ethanol, 4 Jun 2002, Coll. R. Wetzer, S. Boyce, and T. Haney, Collection ID: RW02.021
	MH427772	3115	
	MH427773	3116	
	MH427761	3104	California, Santa Cruz County, San Lorenzo River at Laurel Street bridge, 36.97°N; 122.023°W, freshwater, probably fixed and preserved in 95% ethanol, 22 Mar 2002, Coll. D. Christopher Rogers, Collection ID: RW03.010
	MH427762	3105	
	MH427753	2543	California, Humboldt County, Arcata Bay Margin, mouth of Mad River Slough and tributary at crossing Hwy. 255, ~2 mi. W. of Arcata, ~40.833°N; ~124.133°W, CCS99-69, fixed and preserved in 75% ethanol, 19 Oct 1999, salinity 25°/°°, Coll. Camm Swift, Todd Haney, Dave Jacobs, Collection ID: RW00.009
	MH427759	3102	
	MH427760	3103	
	MH427751	2541	California, Del Norte County, Lake Earl, ~2 mi NNE of Crescent City at end Buzzini Road along E side, salinity 5°/°°, 41.831°N; 124.188°W, probably fixed in 95%, preserved in 70% ethanol, 18 Oct 1999, CCS99-71, Coll. Camm Swift, Todd Haney, Dave Jacobs, Collection ID: RW00.011
	MH427763	3106	
	MH427756	2549	California, Marin County, Walker Creek, US Hwy. 1, ~100 m above mouth of Keyes Creek, 1.5 km SW of Tomales, salinity 1–12°/°°, 38.232°N; 122.912°W, probably fixed in 95%, preserved in 70% ethanol, 21 Oct 1999, Coll. Camm Swift and Todd Haney, Collection ID: RW00.015
	MH427768	3111	
	MH427769	3112	
	MH427752	2542	California, Del Norte County, Smith River, at mouth of Tillas Slough and Rittman Creek at tide gate, ~2 m W of town of Smith River, stream to 30 m, ~41.931°N; ~124.185°W, probably fixed in 95%, preserved in 70% ethanol, 18 Oct 1999, CCS99-70, Coll. Camm Swift, Todd Haney, Dave Jacobs, Collection ID: RW00.010
	MH427764	3107	California, Sonoma County, Salmon Creek at Hwy. 1, ~4.8 km N of N edge of Bodega Bay, salinity 9–23°/°°, ~38.17°N; ~122.28°W, probably fixed in 95%, preserved in 70% ethanol, 19 Oct 1999, CCS99-76, Coll. Camm Swift and Todd Haney, Collection ID: RW00.013
	MH427765	3108	
	MH427774	3117	California, Marin County, Tomales Bay, off Hwy. 1, Alan Sieroty State Park, Millerton Point, ~38.109°N; ~122.851°W, fixed and preserved in 95% ethanol, 4 Jun 2002, Coll. R. Wetzer, S. Boyce, and T. Haney, Collection ID: RW02.022
	MH427775	3118	
*Gnorimosphaeroma noblei*	MH427765	3109	California, Marin County, Schooner Bay at crossing of Sir Francis Drake road to coast of Drakes Bay, 5.5 km W Inverness (airline), salinity 9–23°/°°, 38.232°N; 122.912°W, probably fixed in 95%, preserved in 70% ethanol, 20 Oct 1999, CCS99-82, Coll. Camm Swift and Todd Haney, Collection ID: RW00.014
	MH427767	3110	
	KU248165	1174	California, San Mateo County, San Gregorio Creek, lagoon, just W of US Hwy, stream width 30–40 m, 37.321°N; 122.402°W, fixed and preserved in 75% ethanol, 17 Oct 1999, CCS99-68, Coll. Camm Swift, Dave Jacobs, Todd Haney, Collection ID: RW00.008
	MH427754	2544	
	MH427757	3100	
	MH427758	3101	
Gnorimosphaeroma oregonense*	MH427781	3131	British Columbia, Vancouver, Stanley Park, 49.294°N; 123.155°W, mid intertidal, hand, fixed and preserved in 95% ethanol, 7 Jul 2010, Coll. R. Wetzer & N. D. Pentcheff, Collection ID: RW10.003
*Gnorimosphaeroma oregonense*	AF260866	324	British Columbia, University of British Columbia, ~49.256°N; ~123.257°W, nude, rocky intertidal, among mussels, fixed and preserved in 95% ethanol, 25 Jun 1998, Coll. T. J. Hilbish, Collection ID: RW98.033
	MH427778	3099	
	KU248218	1496	Washington, northeast of San Juan Island, Reuben Tarte County Park, 48.612°N; 123.098°W, underside of rocks in intertidal, hand, fixed and preserved in 95% ethanol, 9 Apr 2004, #7, Coll. R. Wetzer & N. D. Pentcheff, Collection ID: RW04.040
	MH427780	3126	
	KU248217	1151	Washington, westside of San Juan Island, Deadman Bay, 48.513°N; 123.008°W, cobble/sand beach washes, hand, fixed and preserved in 95% ethanol, 8 Apr 2004, #5, Coll. R. Wetzer & N. D. Pentcheff, Collection ID: RW04.038
	MH427779	3125	
	KU248330	1477	Washington, north end of Whidbey Island, Deception Pass, ~48.405°N; ~122.646°W, rocky intertidal among mussels, fixed and preserved in 95% ethanol, 25 Jun 1998, Coll. T. J. Hilbish, Collection ID: RW98.031
	MH427776	3096	
	MH427777	3097	
*Gnorimosphaeroma rayi*	MH427784	2567	California, Marin County, Tomales Bay, north end of bay across from Hog Island, boat launch parking lot, 38.201°N; 122.922°W, intertidal, from underside of rocks, hand, fixed and preserved in 95% ethanol, 9 Jan 2009, #2, Coll. R. Wetzer, Collection ID: RW09.002
	MH427785	2567	
	MH427790	3129	
	MH427786	2568	California, Marin County, Tomales Bay, Marshall, beach in front of Tomales Bay Oyster Company, 15479 Highway One, 38.116°N; 122.854°W, intertidal, from under rocks on sandy beach, hand, fixed and preserved in 95% ethanol, 9 Jan 2009, #1, Coll. R. Wetzer, Collection ID: RW09.001
	MH427787	2568	
	MH427789	3128	
	MH427783	2566	California, Marin County, Tomales Bay, north end of bay across from Hog Island, boat launch parking lot, 38.201°N; 122.922°W, intertidal, from empty *Balanus glandula* testes, hand, fixed and preserved in 95% ethanol, 9 Jan 2009, #2, Coll. N. D. Pentcheff, Collection ID: RW09.006
	MH427791	3130	
	MH427783	2566	
	MH427788	2958	California, Marin County, Bolinas Beach, 37.902°N; 122.686°W, intertidal, hand, fixed and preserved in 95% ethanol, 3 Sep 2009, Coll. Martin Hauser and Darolyn Striley, Collection ID: RW09.072

### Abbreviations

**DISCO** Diversity Initiative of the Southern California Ocean;

**LACM / NHM**Natural History Museum of Los Angeles County;

**MBPC** Marine Biodiversity Center;

**NWU** North-West University;

**PMS** plumose marginal setae;

**RS** robust seta/e;

**SEM** scanning electron microscopy.

Latitudes and longitudes denoted with “~” are approximate and estimated from Google Earth or otherwise estimated and not recorded during specimen collection.

## Results

### Taxonomy

#### Gnorimosphaeroma

Taxon classificationAnimaliaIsopodaSphaeromatidae

Menzies, 1954

F46CEDA4-F412-5DFB-9AD0-4CAD6E0AF58D

Isopoda : Sphaeromatidea: Sphaeromatoidea: Sphaeromatidae
Gnorimosphaeroma Menzies, 1954: 5; Kussakin 1979: 406; Harrison and Ellis 1991: 939.
Nishimuraia Nunomura, 1988: 1.

##### Type species.

*Spheroma oregonensis* Dana, 1853; now *Gnorimosphaeroma oregonense* (Dana, 1853); by original designation.

##### Diagnosis.

*Body* vaulted, dorsal surfaces smooth or polished in appearance, without setae. *Eyes* lateral, simple, without posterior lobe. *Pleon* consisting of 4 visible segments (as determined by lateral sutures), sutures (except first) long extending from lateral margin, separated medially by 24–28% pleon width; pleonite 1 entire, posterior margin even, narrower than remainder of pleon, not extending to pleon lateral margins. *Pleotelson* vaulted, anteriorly as wide as pleon, without dorsal process; posterior margin entire, simple, arcuate. *Maxilliped* palp articles 2–4 medial margins lobate, article 2 not expanded. *Penial processes* entirely separate, basally close set, short (not extending beyond pleopod peduncles). *Uropod* rami lamellar, similar in size, exopod shorter than endopod, inserted near anterolateral angle of peduncle; endopod lateral margin simple, finely serrate or smooth, distally broadly rounded; both rami distally broadly rounded or narrowly rounded.

##### Description.

*Body* vaulted, dorsal surfaces smooth or polished in appearance, without setae; coxal and other margins smooth, with ability to conglobate; not or weakly sexually dimorphic. *Head* with rostral point present, dorsally visible, simple, not separating antennular bases; without paired incisions in front of eyes, lateral margins not laterally extended to body outline (antennules more or less ventral). *Eyes* lateral, simple. Pereonite 1 lateral margins not anteriorly produced, not laterally enclosing head, pereonites 2–7 with posterior margin not raised, pereonite 1 anteriorly with keys. *Sternite 1* without cuticular mesial extensions. *Pereonite 6* simple, without bosses, processes or marginal extensions. *Pereonite 7* as wide as pereonite 6, forming part of body outline, dorsally without bosses, processes, or marginal extensions. *Coxae* distally narrow, those of pereonites 2–7 overlapping the one behind, rounded, with ventral ‘lock and key’ processes, with grooved articulation; those of pereonite 6 not large, not overlapping those of pereonite 7. *Pleon* consisting of 4 visible segments (as determined by lateral sutures); pleonite 1 entire, posterior margin even, narrower than remainder of pleon, not extending to pleon lateral margins; sutures (except first) running to lateral margin, all separate, sutures long (separated medially by 24–28% pleon width); pleonal sternite absent; dorsal surface without process; posterior margin even, with ‘keys’. Pleonite 5 posterior margin entire (not fused with pleotelson). *Pleotelson* vaulted, anteriorly as wide as pleon, without dorsal process; posterior margin entire, simple, arcuate; ventrolateral margins forming ridge.

*Marsupium* formed from four pairs of oostegites, arising from pereonites 1–4; anterior pocket absent, posterior pocket absent, oostegites overlapping at mid-line (except 1).

*Antennule* peduncle with basal articles medially not in contact, 1 and 2 robust, article 3 slender; article 1 not produced, without anterior lobe; article 2 approximately 0.5 as long as article 1; with articles 2 and 3 colinear, article 3 longer than article 2; article(s) not flattened; flagellum shorter than peduncle, longer than peduncular article 3. *Antenna* peduncle articles all colinear (or curving regularly), less robust than antennule, peduncular articles all of similar thickness.

*Epistome* anteriorly narrow, with median weak constriction, anteriorly flush with head, not projecting; elongate. *Mandible* incisor wide, 4-cuspid; lacinia mobilis present; spine row normal; present, molar process gnathal surface with transverse ridges, rounded. *Maxillula* lateral lobe robust setae with some or all serrate, mesial lobe with major robust setae, these setae being heavily serrate. *Maxilla* with setae on middle and lateral lobes serrate. *Maxilliped* palp articles 2–4 medial margins lobate, article 2 not expanded; endite distal margin rounded, anteromesial (upper) marginal ridge without long curved serrate robust setae.

*Mouthparts of female* not metamorphosed.

*Pereopod 1* ambulatory; dactylus secondary unguis short, robust, simple; setae on superodistal corner of merus only very long. Pereopod 2 similar in proportion to pereopod 3; dactylus with secondary unguis simple, short and stout. Pereopods 3–7 dactylus with secondary unguis simple. Pereopods with inferior margins of ischium to carpus without dense setulose fringe, ischium superior margin without sinuate acute robust seta, pereopods 1–3 or 4 ischium superior margin with few long stiff slender setae. Pereopods 1 (or 1–3), inferior margins of merus, carpus and propodus palm pereopod 1 only with robust setae on propodus inferior margin.

*Penial processes* entirely separate, basally close set, short (not extending beyond pleopod peduncles), widest near base, apex bluntly rounded.

*Pleopod 1* rami not operculate; exopod lamellar; rami exopod with longitudinal axis weakly oblique; endopod of similar proportions to exopod, mesial margin lamellar, distally triangular, endopod proximomedial heel absent; exopod distally rounded or distally subtruncate or truncate, exopod distal margins not serrate. *Pleopod 2* endopod ca. as long as exopod; exopod distal margins not deeply serrate; appendix masculina inserted basally, with straight margins, distally abruptly narrowed, longer than and extending beyond endopod (1.14 × as long as endopod), distally narrowly rounded. *Pleopod 3* exopod transverse suture present, endopod of similar proportions to exopod. *Pleopod 4* rami with PMS; exopod transverse suture present, incomplete, thickened transverse ridges absent, lateral margin not thickened, with short simple marginal setae; endopod thickened transverse ridges absent; mesial margin without deep distal notch; endopod without proximomedial lobe. *Pleopod 5* exopod transverse suture present, entire, thickened transverse ridges absent, lateral margin with short simple setae, lateral margin not thickened, with 3 discrete scale patches; scale patches flush or weakly domed; endopod with thickened transverse ridges absent, endopod without proximomedial lobe.

*Uropod* rami not strongly flattened, not forming part of continuous body outline; exopod shorter in length than endopod, exopod lamellar, inserted near anterolateral angle of peduncle-endopod, lateral margin simple, finely serrate or smooth, distally broadly rounded; endopod lamellar, distally broadly rounded or narrowly rounded. Uropod endopods not in contact posteriorly.

##### Remarks.

*Gnorimosphaeroma* is in a general sense quite unremarkable in appearance, with no species showing any sort of dorsal ornamentation of tubercles, processes, or pereonal and pleonal ridges that characterize so many genera of Sphaeromatidae. As such, there is a lack of readily obvious characters by which to identify the genus. *Gnorimosphaeroma*, on morphological criteria, is most similar to the genera *Bilistra* Sket & Bruce, 2004, *Exosphaeroma* Stebbing, 1900, *Lekanesphaera* Verhoeff, 1943, *Neosphaeroma* Baker, 1926 and *Sphaeroma* Bosc, 1802. The latter three genera can be differentiated from *Gnorimosphaeroma* in the first instance by having the uropodal exopod lateral margin with one or more serrations or notches (among other characters).

*Exosphaeroma* is a large genus with 40 species at the last count (Boyko et al. 2008) that, as presently constituted, contains both smooth bodied species as well as some with coarsely pitted or ridged dorsal surfaces (e.g., see Kensley 1978; Espinosa-Pérez and Hendrickx 2001; Bruce 2003), and also species with greatly enlarged uropodal rami (e.g., see Kensley 1978; Bruce 2003; Wall et al. 2015). Some of the smooth-bodied species of *Exosphaeroma* are superficially similar to *Gnorimosphaeroma*, but can be distinguished by the pleonal sutures running to the posterior margin (to the free lateral margin in *Gnorimosphaeroma*), as well as pleonite 1 having two flat sub-median lobes on the posterior margin (see Bruce 2003: figs 14E, 18F).

*Bilistra* is similar in gross morphology and also occupies coastal freshwater habitats. *Bilistra* differs from *Gnorimosphaeroma* in having a far shorter uropodal exopod (ca. half as long as endopod), shorter pleonal sutures that run to the pleon posterior margin (not lateral margin); the inferior margins of pereopods ischium or merus to propodus have a dense setulose (fur-like) fringe while the superior margins lack long setae altogether. *Bilistra* is presently restricted to New Zealand, but there is also one species in South Africa, from supralittoral brackish pools and tidal streams that is currently classified as *Pseudosphaeroma barnardi* Monod, 1931 that is in need of redescription and formal reassignment to *Bilistra* (NLB, pers. obs.).

*Gnorimosphaeroma* pereopod setation is inconsistently illustrated, even within species, despite being a potentially significant character. The redescription given here, and figures of Hoestlandt (1975) show long setae on the superior or superodistal margin of the merus and long setae on the inferior margin of the ischium and merus. Such setae were not mentioned or figured in Menzies’ (1954) genus diagnosis or species descriptions. Such setae are also apparently absent from all northwestern species (e.g., Hoestlandt 1975, 1977; Kwon and Kim 1985; Nunomura 1998, 1999a, 2007).

##### Neotype designation.

It has been long established that all of Dana’s (1852) isopod material, and therefore all the type material for the many species of isopod that he named, was lost with the sinking of the ship USS ‘Peacock’ on the bar of the Columbia River in 1841 (Bruce 1986: 220; 2004: 228; 2009: 211; Poore and LewTon 1993: 234). *Gnorimosphaeroma oregonense* (Dana, 1853) is one such species.

Species of *Gnorimosphaeroma* are uniform in appearance, and to date no assessment has been made of intrinsic variability within species. Some species of *Gnorimosphaeroma* occur sympatrically and there are many exceedingly similar species. At present few species have been described in full detail. Furthermore, records of *G. oregonense* are somewhat inconsistent in the details presented and the material is not always available for re-examination, so that it is not always possible to confirm the correct identity of previous records and indeed also on occasion, new material. We consider that designating a neotype is necessary to clearly characterize the identity of this species, to allow for the genus to be precisely diagnosed based on the type species and to permit unambiguous identification and separation from other sympatric congeneric species.

Dana (1853) did not indicate a specific type locality, but stated that the species had been obtained from “Puget’s Sound, Oregon; also, Bay of San Francisco, California”. One may infer that the first mentioned location is the type locality but that remains an inference, and furthermore one cannot be certain that the material consists of only one species, given that there are four species in the region and also that the morphology of purported species apparently changes from low to high latitudes (present study). The neotype has been chosen from specimens collected as near as practically possible to the original type locality, and is now Stanley Park, 49.294°N, 123.155°W (British Columbia, Canada), ca. 150 km north of Puget Sound.

##### Included species.

*Gnorimosphaeroma albicauda* Nunomura, 2005, *G. akanense* Nunomura, 1998, *G. anchialos* Jang & Kwon, 1993, *G. boninense* Nunomura & Satake, 2006, *G. chejuense* Kim & Kwon, 1988, *G. chinense* (Tattersall, 1921), *G. hachijoense* Nunomura, 1999b, *G. hoestlandti* Kim & Kwon, 1985, *G. hokurikuense* Nunomura, 1998, *G. insulare* (Van Name, 1940), *G. iriei* Nunomura, 1998, *G. kurilense* Kussakin, 1974, *G. naktongense* Kwon & Kim, 1987, *G. noblei* Menzies, 1954, *G. oregonense* (Dana, 1853), *G. ovatum* (Gurjanova, 1933), *G. paradoxa* (Nunomura, 1988), *G. pulchellum* Nunomura, 1998, *G. rayi* Hoestlandt, 1969, *G. rebunense* Nunomura, 1998, *G. saijoense* Nunomura, 2013, *G. shikinense* Nunomura, 1999b, *G. tondaense* Nunomura, 1999b, *G. trigonocaudum* Nunomura, 2011, *G. tsutshimaense* Nunomura, 1998.

##### Notes.

The original diagnosis of the genus was provided by Menzies (1954: 5). A more complete diagnosis of the genus is provided here (see above). Menzies (1954) suggested that *Neosphaeroma pentaspina* Baker, 1926 could possibly be attributed to *Gnorimosphaera* were it to be redescribed, while Harrison and Holdich (1984) indicated some shared characters, notably the pleon suture, but the species is presently considered as *incertae sedis*. Smooth-bodied Sphaeromatidae similar to *Gnorimosphaeroma* are summarized in the genus remarks above and reoccur in several sphaeromatid clades. In their molecular analysis Wetzer et al. (2018) demonstrated that this a plesiomorphic trait and that *Neosphaeroma* is basal to or nested within the *Cymodoce* clade and is not closely related to *Gnorimosphaeroma*.

#### Gnorimosphaeroma oregonense

Taxon classificationAnimaliaIsopodaSphaeromatidae

(Dana, 1853)

0BC8E461-C1CD-53B2-B84F-2D40FA30A3A1

Figures 1 , 2 , 3 , 4 , 5 , 6 , 7 , 8 , 9

Abbreviated synonymy (detailed synonymies given by Richardson (1905), Menzies (1954), and Kussakin (1979).
Spheroma oregonensis Dana, 1853: 778, Atlas plate 52x.
Exosphaeroma oregonensis .— Richardson, 1905: 296, figs 315, 316.
Neosphaeroma oregonense .— Monod, 1932: 76, fig. 74.
Gnorimosphaeroma oregonensis oregonensis .— Menzies, 1954: 406, figs 5, 7A–E, 12.

##### Material examined.

***Neotype*** ♂ (8.5 mm): Canada, British Columbia, Vancouver, Stanley Park, 49.294°N, 123.155°W, mid intertidal, hand, fixed and preserved in 95% ethanol. 7 Jul 2010, coll. Regina Wetzer & N. Dean Pentcheff. Collection ID: RW10.003. LACM:DISCO:7028.

##### Additional material examined from the same lot as the neotype.

♀ Non-type with mancas (6.0 mm) LACM:DISCO:11164; ♂ (8.5 mm) LACM:DISCO:11161; subadult ♂ with penes beginning, without appendix masculina (6.0 mm) LACM:DISCO:11162; plus additional 20+ adults, juveniles, and mancas in this lot.

Body parts and appendages figured are as indicated in figure legends.

##### Description of male neotype.

**Figure 1.**
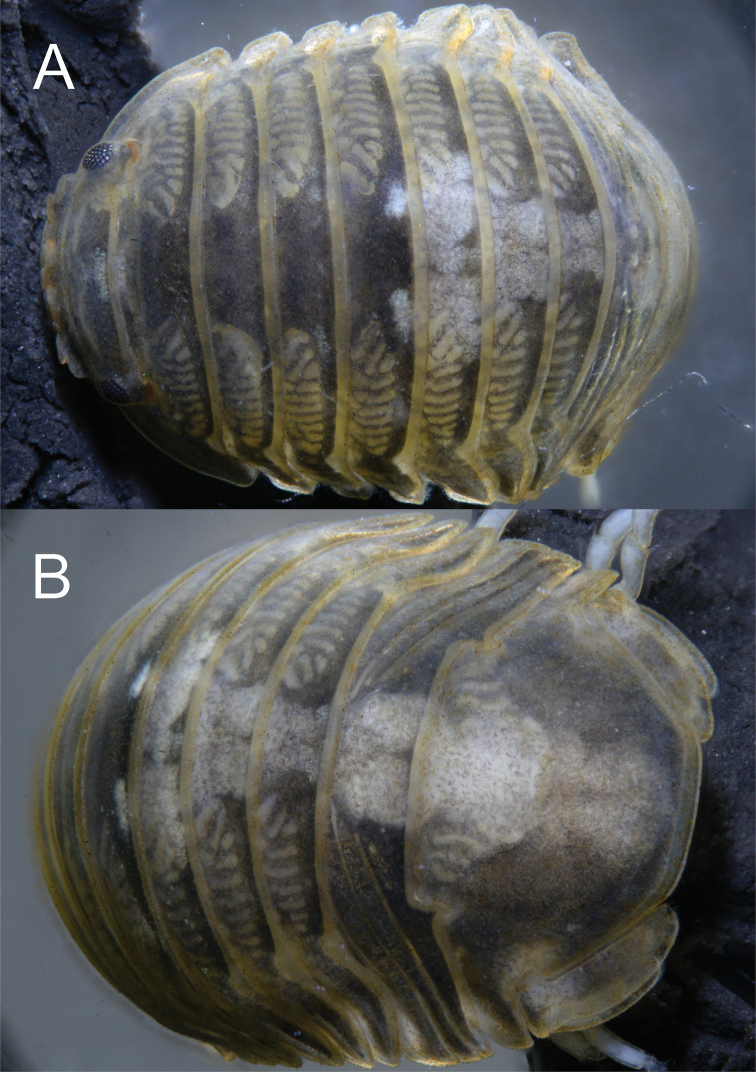
*Gnorimosphaeroma oregonense*. ♂ Neotype. LACM:DISCO:7028 **A** anterior dorsal **B** posterior dorsal and pleotelson.

**Figure 2.**
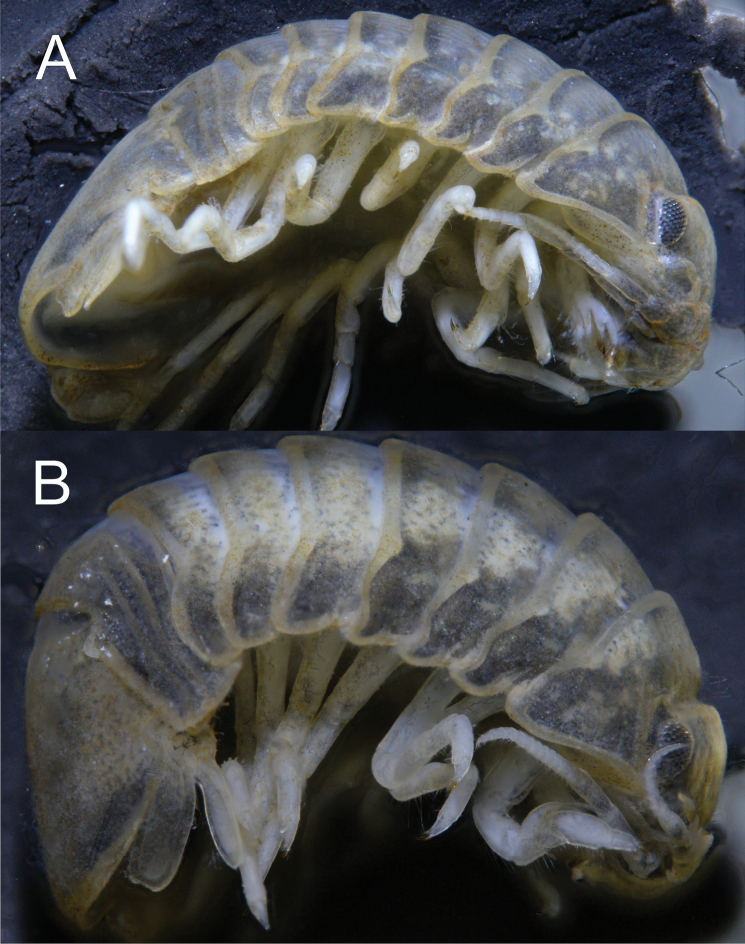
*Gnorimosphaeroma oregonense***A** ♂ neotype. LACM:DISCO:7028, lateral **B** ♀ non-type LACM:DISCO:11164, lateral.

*Body* length 2.4 × width; widest at pereonite 6; pleotelson length 0.6 × width, distal margin broad and weakly convex. (Figs 1A, B, 2A). *Pleotelson* length 0.66 × width.

**Figure 3.**
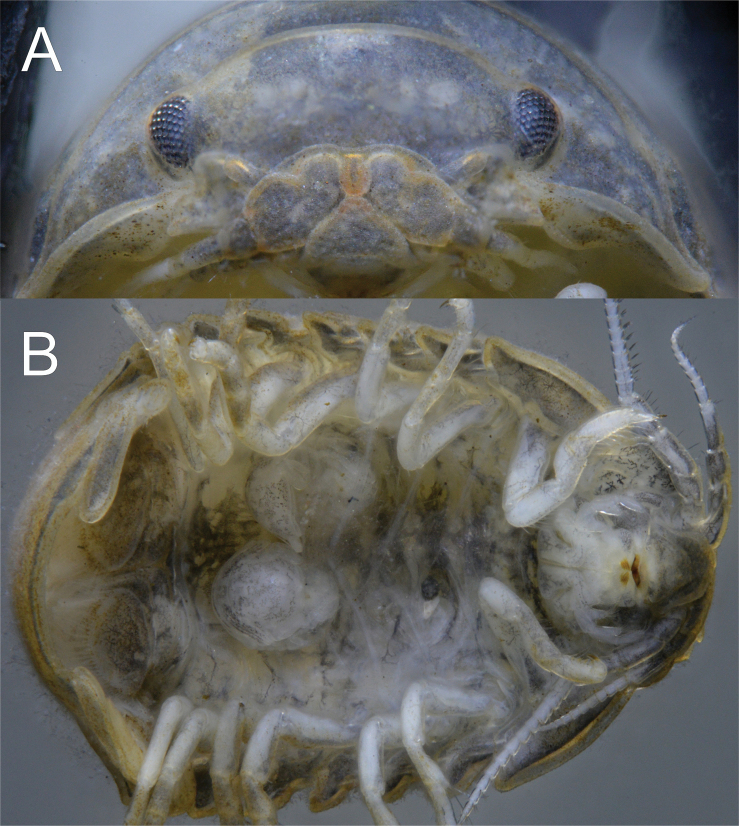
*Gnorimosphaeroma oregonense* ♀ Non-type. LACM:DISCO:11164 **A** clypeus and labrum **B** marsupium with three mancas.

**Figure 4.**
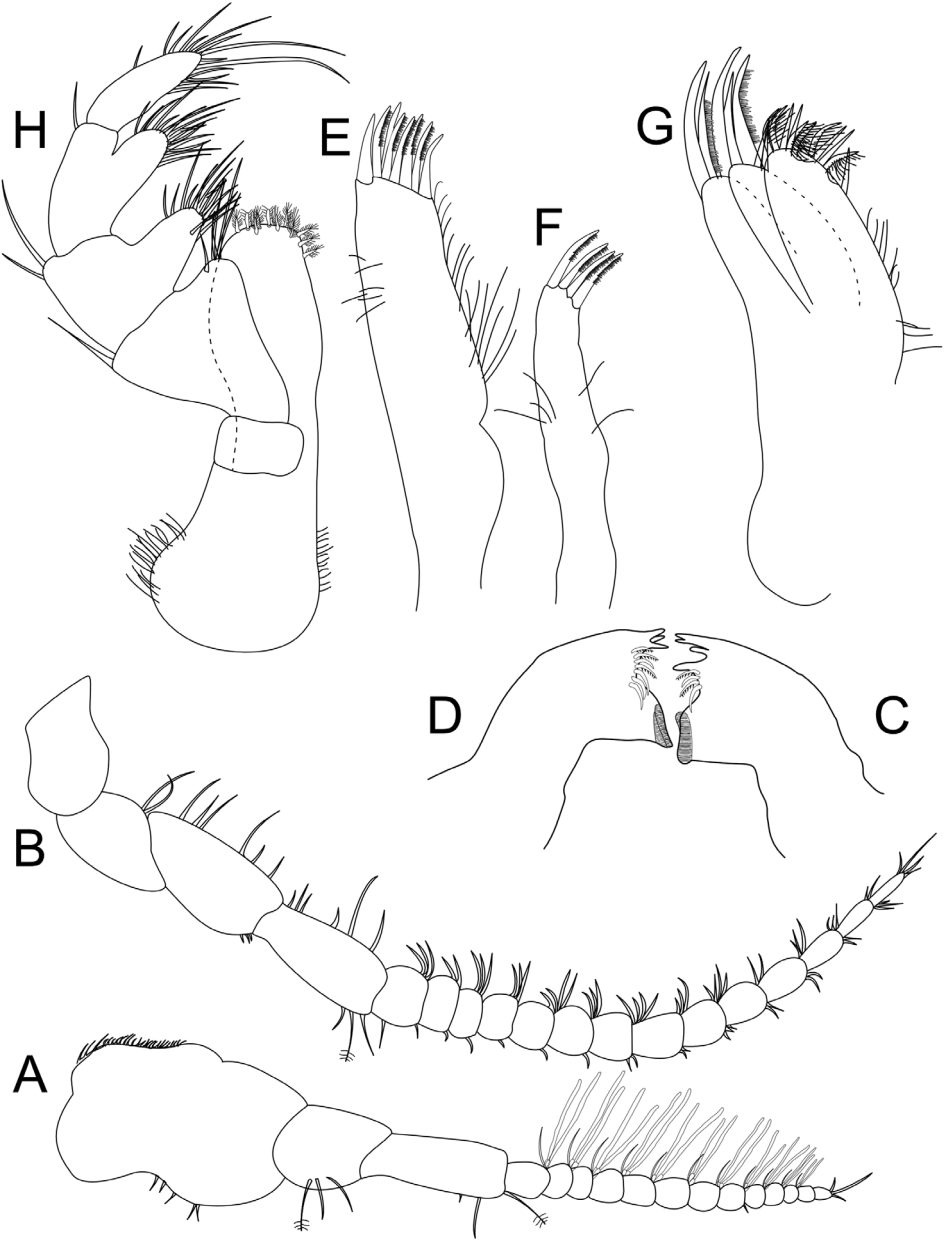
*Gnorimosphaeroma oregonense* ♂ Neotype LACM:DISCO:7028. All appendages from right, unless otherwise indicated **A** antennula **B** antenna **C** left mandible **D** right mandible **E** maxillula lateral lobe **F** maxillula medial lobe **G** maxilla **H** maxilliped.

*Antennula* peduncle article 1 length 1.3 × width; article 2 as long as wide; article 3 length 2.6 × width, inferior distal margin with one palm seta; flagellum with 13 articles, 11 basal articles with aesthetascs and small simple seta (Figs 2A, 3A, 4A). *Antenna* reaching slightly beyond anterior margin of pereonite 2; peduncle article 4 length 2.3 × width, flagellum with 14 articles, setation as figured (Figs 2A, 3B, 4B). *Clypeus* and *labrum* as in Figs 3A, 8B.

*Left mandible* incisor with 4 cusps; lacinia mobilis with a single cusp; lacinia mobilis spine row comprised of 4 serrate spines; crushing surfaces ridged (Fig. 4C). *Right mandible* incisor with 3 cusps, spine row comprised of 7 serrate spines (Fig. 4D). *Maxillula* mesial lobe with ca. 4 spines; lateral lobe with ca. 8 spines (Fig. 4F, E, respectively). *Maxilla* mesial lobe with 5 simple setae and 6 plumose RS on gnathal surface; middle lobe with 2 simple setae and 1 pectinate RS; lateral lobe with 2 simple setae, and 1 pectinate RS (Fig. 4G). *Maxilliped* endite distal surface with 7 plumose setae; distomesial margin with 3 plumose setae; palp article 2 distal apex with 9 long, simple RS; article 3 distal apex with 11 long, simple RS, lateral distal angle with 2 long, simple RS; article 4 distal apex with 15 long, simple RS, lateral distal angle with 1 long, simple RS; article 5 distal apex with 13 long, simple RS (Fig. 4H).

**Figure 5.**
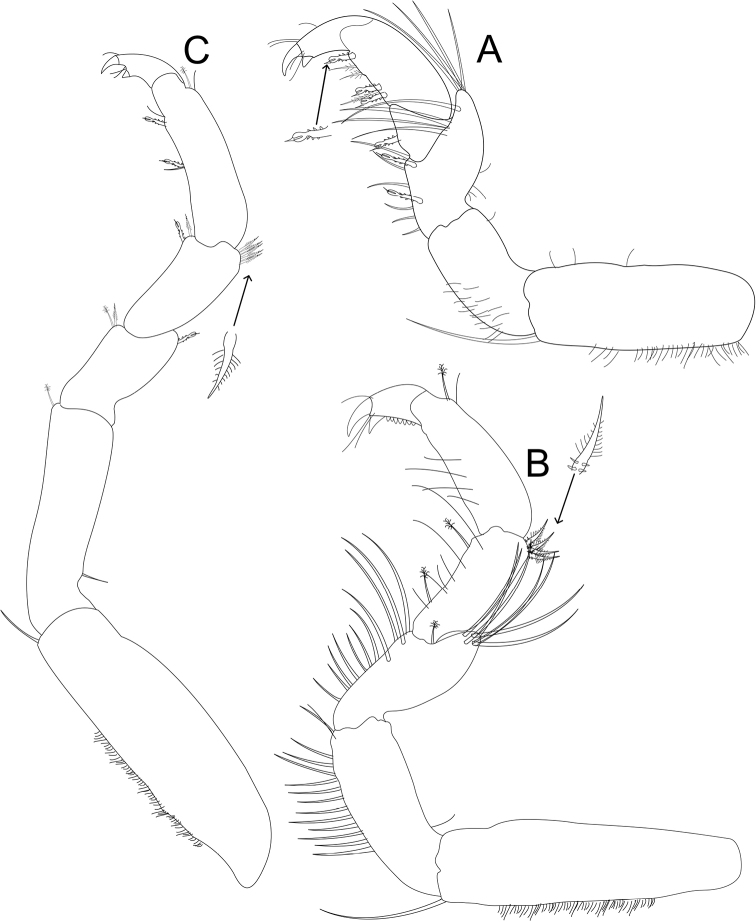
*Gnorimosphaeroma oregonense* ♂ Neotype LACM:DISCO:7028. All appendages from right **A** pereopod 1 **B** pereopod 2 **C** pereopod 7.

*Pereopod 1* (Figs 5A, 7C) *basis* inferior distal angle with 1 long, RS, inferior proximal margin with setal patch; *ischium* length 1.6 × width, inferior medial margin with setal patch; *merus* lobate, 0.74 × ischium length, superior distal angle with 4 long, RS; *carpus* inferior medial margin with 1 robust, serrate, trident seta; *propodus* length 2.1 × width, 1.1 × ischium length, inferior margin with 3 robust, serrate, trident seta, and 3 plumose setae; *dactylus* length 1.2 × width, length 0.33 × propodus length, distal margin with 4 simple setae (Figs 5A, 7C). *Pereopod 2* (Fig. 5B) *basis* inferior distal angle with 1 long, simple RS, inferior medial margin with setal patch; *ischium* length 2.2 × width, inferior medial margin with 12 long, simple RS, inferior distal angle with single simple RS; *merus* lobate, length 1.6 × width, 0.69 × ischium length, superior distal angle with cluster of 7 simple RS, distal medial margin with one palm seta; *carpus* length 1.2 × merus length, 2.5 × width, superior margin with 4 robust, biserrate setae on distal angle, inferior margin 2 palm setae; *propodus* weakly curved, length 2.6 × width, 1.2 × carpus length, superior distal margin with a palm seta; *dactylus* length 1.2 × width, length 0.27 × propodus length, inferior margin with scales, distal margin with 3 long, simple setae (Fig. 5B). *Pereopods 3–6* progressively less setose (not figured). *Pereopod 7* (Figs 5C, 7B) *basis* inferior medial margin with setal patch, inferior distal angle with 1 long, simple seta; *ischium* length 3.2 × width, inferior distal angle with 1 palm seta; *merus* lobate, merus length 1.3 × width, merus length 0.42 × ischium length, superior distal angle with 1 trident seta, inferior distal angle with 1 biserrate seta and 1 palm seta; *carpus* length 1.8 × width, carpus length 1.3 × merus length, superior distal angle with a cluster of 5 long, biserrate setae, inferior distal angle with a cluster of 1 long, biserrate seta, and 1 long, trident seta; *propodus* weakly curved, length 3.2 width, length 1.5 carpus length, superior distal angle with 1 simple seta, and 1 palm seta, inferior margin with 2 long, trident setae; *dactylus* length 1.3 × width, dactylus length 0.21 × propodus length, distal margin with 3 simple setae (Figs 5C, 7B).

**Figure 6.**
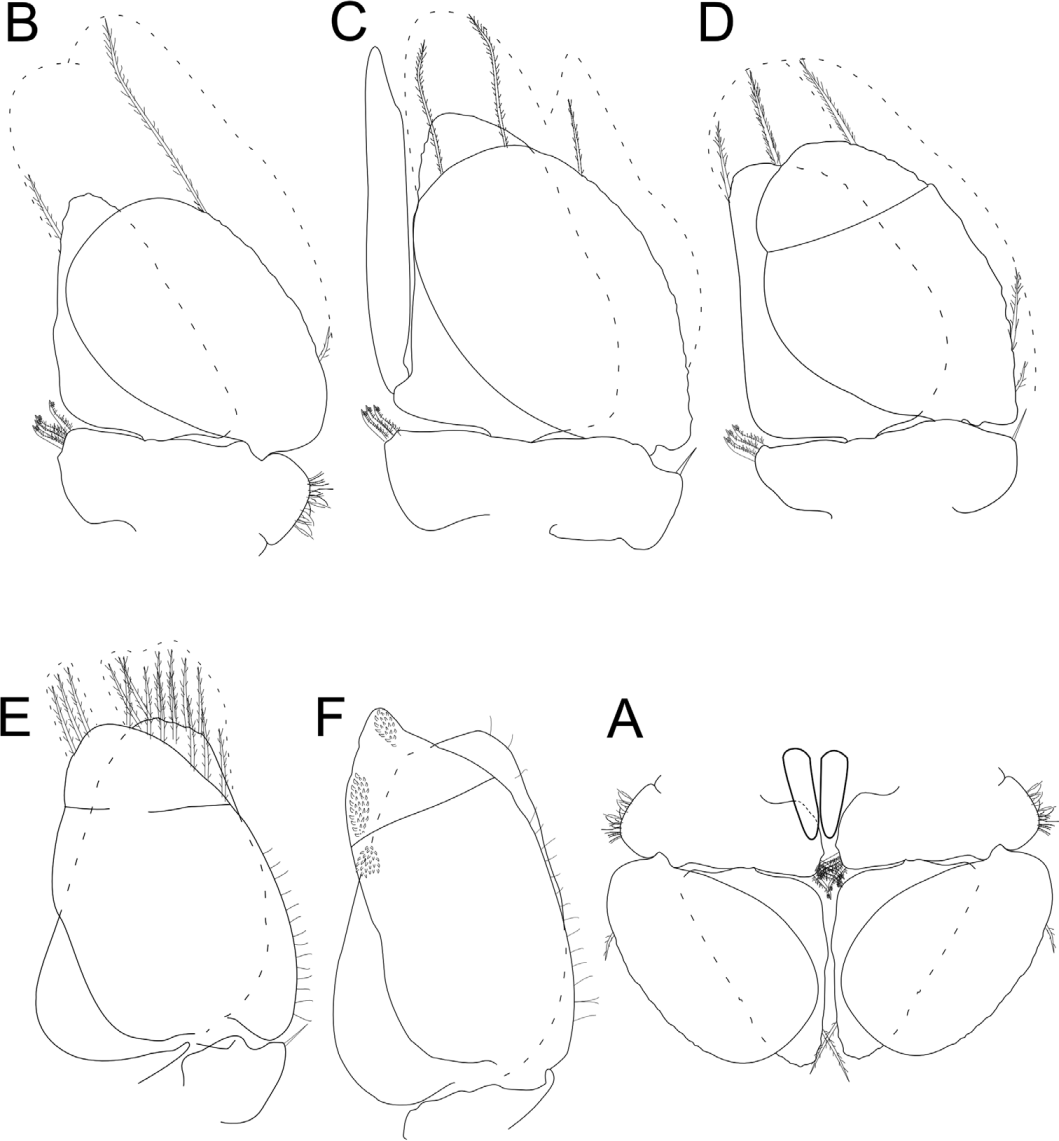
*Gnorimosphaeroma oregonense* ♂ Non-type LACM:DISCO:11161 **A** penes in ventral view with relative position to pleopods. ♂ Neotype. LACM:DISCO:7028. All appendages from right **B–F** pleopods 1–5.

*Penial processes* length 3.8 × basal width; close set (Fig. 6A).

*Pleopod 1* (Fig. 6B) peduncle length 0.38 × width with 4 coupling hooks; exopod length 1.5 × width, 1.1 × endopod length. *Pleopod 2* (Fig. 6C) peduncle length 0.34 × width with 3 coupling hooks, *appendix masculina* length 8.5 × width, 1.1 × length of endopod, straight, proximally and medially slightly swollen, distally narrowing. *Pleopod 3* (Fig. 6D) peduncle length 0.34 × width with 3 coupling hooks. *Pleopods 1–4* exopods and endopods with PMS as figured (note: not all drawn, but indicated). *Pleopod 4* (Fig. 6E) endopod and exopod subequal, exopod with transverse suture. *Pleopod 5* (Fig. 6F) endopod and exopod subequal, endopod length 1.5 × width, exopod length 2.1 × width with 1 distal scale patch and 2 medial lateral scale patches.

*Uropod* extending to posterior margin of pleotelson. *Exopod* 0.83 × as long as endopod, 2.7 × as wide; apex narrowly rounded; mesial margin with continuous row of PMS. *Endopod* 3.8 × as long as wide, lateral margin weakly convex, apex bluntly rounded.

##### Description of female.

**Figure 7.**
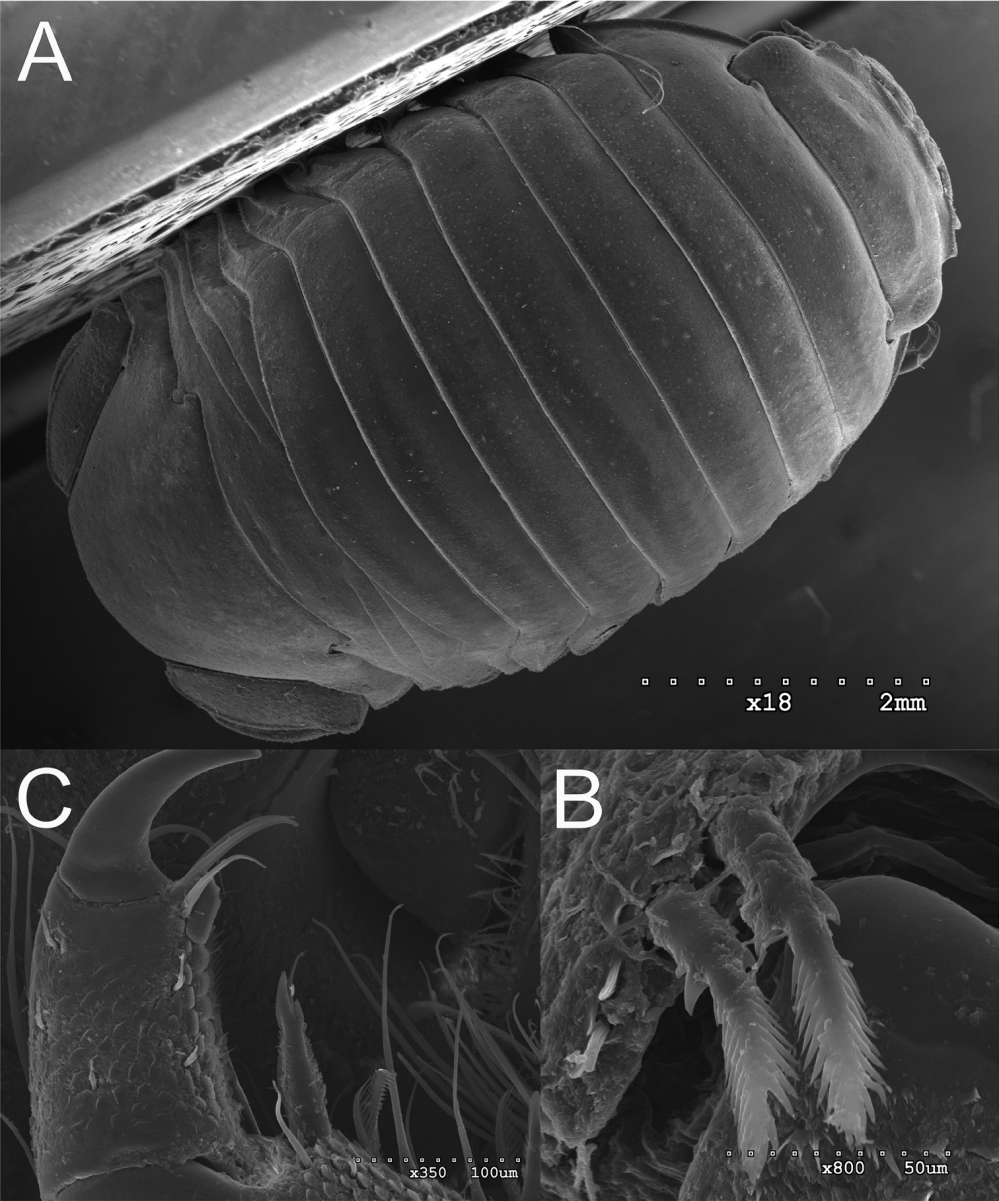
*Gnorimosphaeroma oregonense* ♀ Non-type SEM. LACM:DISCO:11164 **A** dorsum **B** pereopod 1 seta **C** pereopod 7 setae.

**Figure 8.**
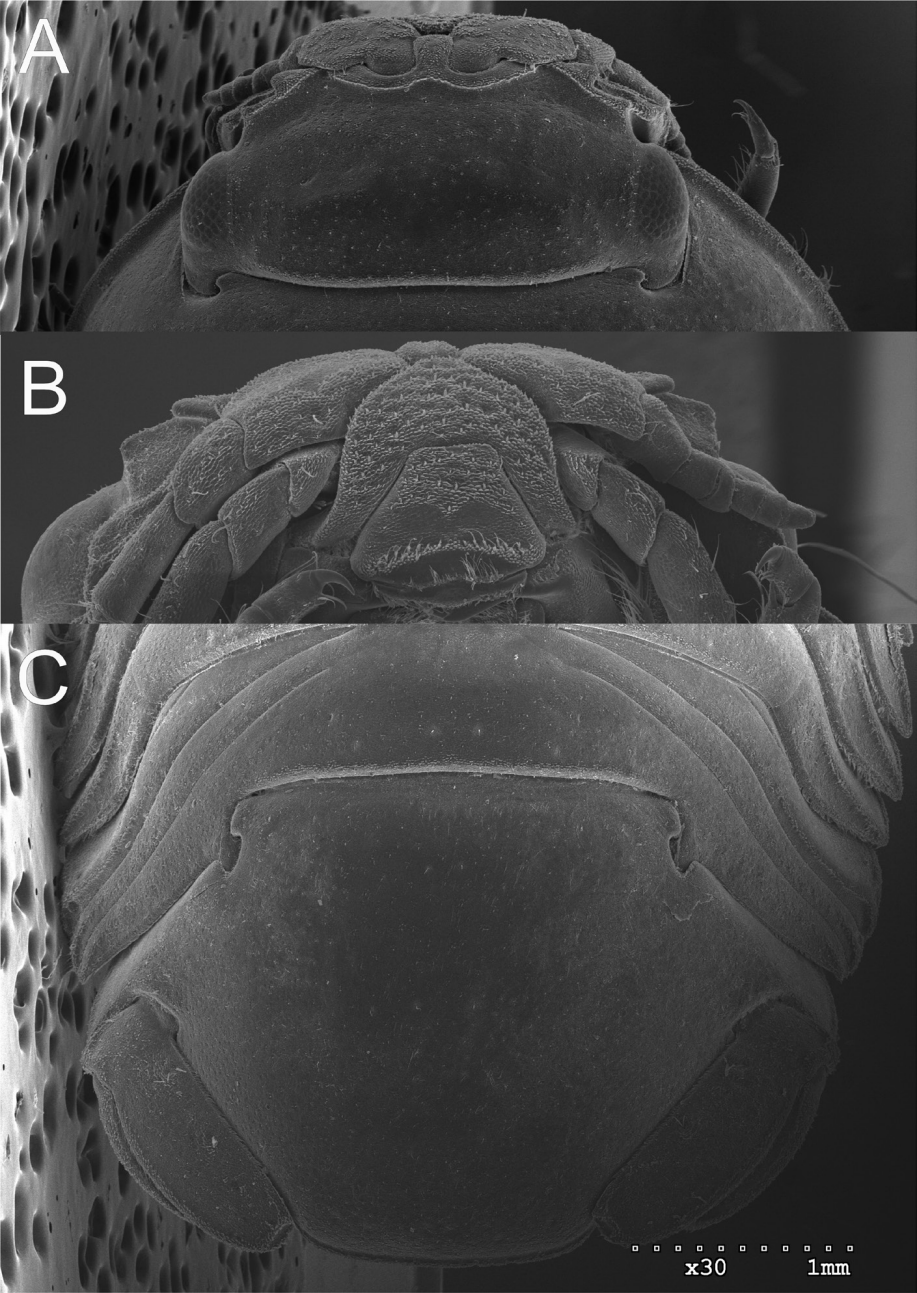
*Gnorimosphaeroma oregonense* ♀ Non-type SEM. LACM:DISCO:11164 **A** head dorsum **B** clypeus and labrum ventral **C** pleotelson dorsal.

**Figure 9.**
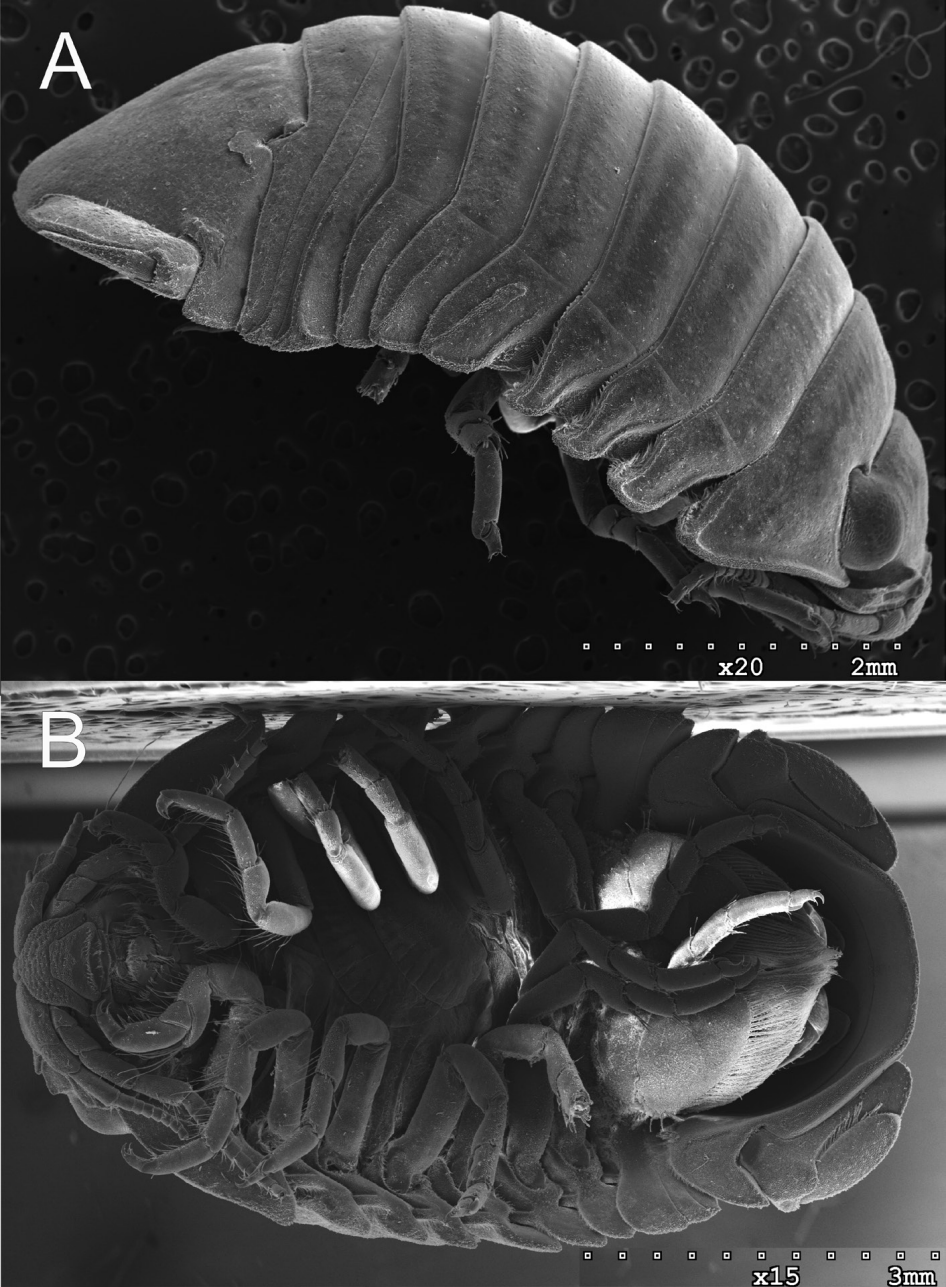
*Gnorimosphaeroma oregonense* Non-type SEM. LACM:DISCO:11164 **A** ♀ LACM:DISCO:11164 lateral **B** ♀ LACM:DISCO:11164 ventral view with oostegites.

*Body* length 2.4 × width (Figs 2B, 3A, B, 7A, 8A–C, 9A, B). *Pleotelson* length 0.66 × width (Fig. 8C). *Uropodal* endopod (Figs 8C, 9B) as in male, longer than exopod, endopod just barely extending to posterior margin of pleotelson. Gravid female (Figs 3B, 9B) estimated to be able to brood 8–10 mancas.

##### Size.

Largest ♂ to 8.5 mm, largest ♀ to 6 mm. Dana (1853) gave no measurements. Fee (1926: 8, 9) records the largest specimens as being “ca. 1 cm. long; one-half as long as wide.”

##### Color.

When preserved in ethanol, specimens quickly become pale buff to whitish.

##### Distribution.

British Columbia, Vancouver to California, San Francisco.

##### Remarks.

The species occurs only in fully marine habitats in the intertidal to an unknown depth. A single lot indicated that it was collected by night light, and another that specimens were collected on floats among fouling organisms. None of the material examined indicates depth.

Kussakin (1979) reported *G. oregonense* from Alaska, Popov Island to San Francisco Bay, California. Kussakin (1979) figured *G. oregonense* from the collections of the Zoological Institute of the Academy of Sciences of the USSR. He noted that it is widely distributed with males reaching a length of 12 mm and females up to 8 mm, and that it occurred widely from Alaska to California. It is not clear what the specific localities of the figured specimens were (Kussakin 1979: 407) nor of those deposited in the Russian collections. We were unable to locate and access these specimens. Kussakin reported that the specimens he examined were predominantly littoral, but can be sublittoral to 22 m, on rocks, under rocks, less often on sand, and sometimes in empty shipworm tubes. Kussakin remarked it is a good swimmer, and sometimes turns up in night light samples. It can tolerate salinities as low as 9‰. Since we were not able to re-examine Kussakin’s specimens, we cannot verify that the *Gnorimosphaeroma* he identified are the same species as *G. oregonense* from the type locality and described here. Furthermore, our genetic data clearly distinguishes between fully marine and low salinity specimens and recognizes these as distinct species (see below). We do not include Kussakin’s specimens in the synonymy (*Gnorimosphaeroma oregonense*: Kussakin, 1979: 406, figs 260–262.)

### Molecular analysis

**Figure 10.**
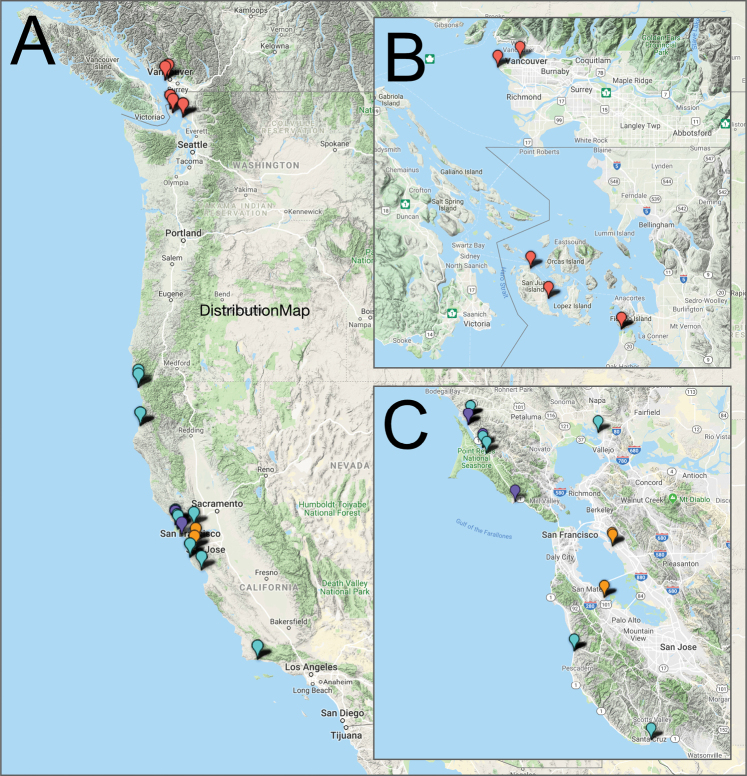
**A** West Coast distribution of *Gnorimosphaeroma* for which genetic material was available. Red = *G. oregonense*, light blue = *G. noblei*, purple = *G. rayi*, orange = *Gnorimosphaeroma* sp. **B***Gnorimosphaeroma oregonense* distribution in Puget Sound for which genetic material was available **C***Gnorimosphaeroma noblei*, *G. rayi*, and *Gnorimosphaeroma* sp. Distribution in San Francisco Bay region.

**Figure 11.**
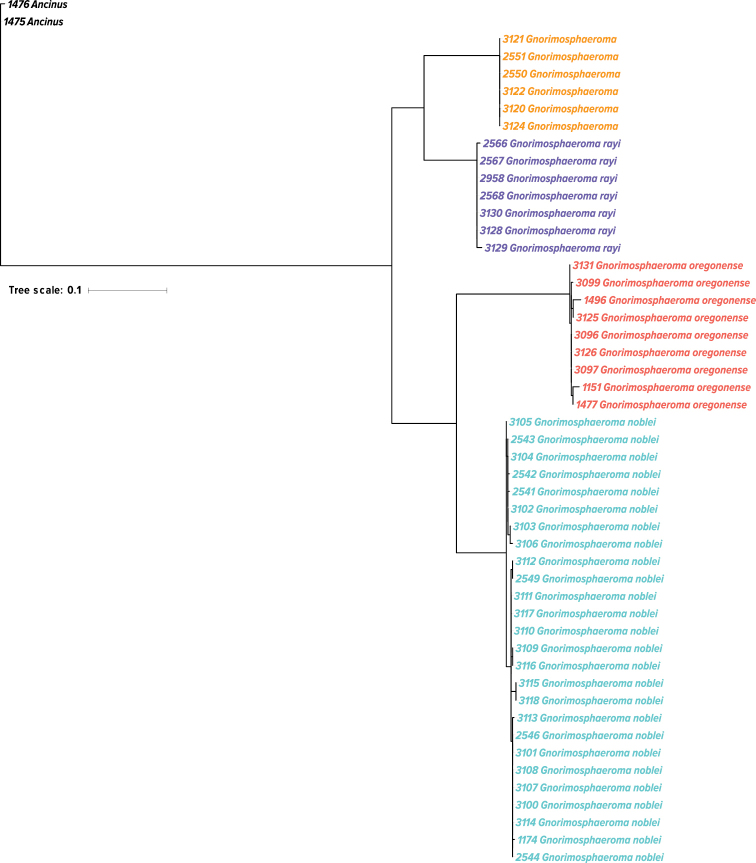
*Gnorimosphaeroma* 16SrDNA phylogeny based on maximum likelihood and 54 sequences. *Gnorimosphaeroma oregonense* (6 localities), *G. noblei* (11 localities), *G. rayi* (4 localities), and 2 localities within San Francisco Bay for the unidentified *Gnorimosphaeroma* sp. Red = *G. oregonense*, light blue = *G. noblei*, purple = *G. rayi*, orange = *Gnorimosphaeroma* sp. (same color coding as in Fig. 10).

The molecular analyses include *G. oregonense* from Vancouver and the San Juan Islands, Washington (49.256°N–48.513°N). There are no specimens north of Vancouver in our collections. *Gnorimosphaeroma noblei* material came from Del Norte to Santa Barbara Counties (40.833°N–34.46°N), *G. rayi* from Marin County (38.201°N–37.902°N), and the unidentified *Gnorimosphaeroma* sp. were collected only in San Francisco Bay (San Mateo and Alameda Counties, latitude 37.079°N–37.535°N). Figure 10 indicates the localities of the sequenced material. Alignment differences resulting from the LINS, EINS, or GINS alignment algorithms had insignificant effect on RAxML and Fasttree analyses and the phylogenetic hypotheses. *Ancinus* sp. (Sphaeromatoidea: Ancinidae) was used as the outgroup based on the basal position of *Gnorimosphaeroma* within the Sphaeromatidae (Wetzer et al. 2013). Both analyses resulted in the same 1 tree. Only the RAxML tree (Fig. 11) is shown.

Our molecular analyses (Fig. 11) clearly distinguish *G. oregonense* and *G. noblei*. They are always sister taxa. *Gnorimosphaeroma rayi*, is always sister to an unidentified *Gnorimosphaeroma* species collected from the two localities in San Francisco Bay.

#### *Gnorimosphaeroma noblei* Menzies, 1954

*Gnorimosphaeroma noblei* Menzies, 1954 was described from the town of Marshall in Tomales Bay, California (~38.162°N, ~122.89°W). Hoestlandt (1969) synonymized *G. oregonense lutea* with *G. noblei*. Menzies noted the species was associated with the terrestrial isopod *Armadilloniscus* in the upper intertidal, and that they were excellent swimmers. This association indicates likely freshwater input and possible lower salinity. This species has the largest range of all of the *Gnorimosphaeroma* species studied here (California, Del Norte County, ~41.931°N to Los Angeles County, 33.802°N). This species also has the broadest salinity tolerance – brackish to freshwater, a characteristic found in only a few sphaeromatid genera. *Gnorimosphaeroma noblei* has been collected from a full range of high intertidal, brackish to fully freshwater habitats including Sacramento, central San Joachin Delta, ~38.33°N, ~121.3°W collections by Wayne Fields. Fields’ specimens were preserved in formalin and their collection date is unknown. They have been in the LACM collections since before 2003. Specimens of *G. noblei* can be comparable in size to *G. oregonense*, but more commonly are slightly smaller. *Gnorimosphaeroma noblei* is purported to occur as far south as Los Angeles County, Dominguez Channel, 33.802°N, 118.228°W. Three very small specimens from 4 m depth were collected 17 September 2003 (MBPC 10592, Collection ID: RW17.028). These were also preserved in formalin and were unavailable for genetic analysis, but based on all of the other material examined (Table 2) are presumed to be *G. noblei*.

**Figure 12.**
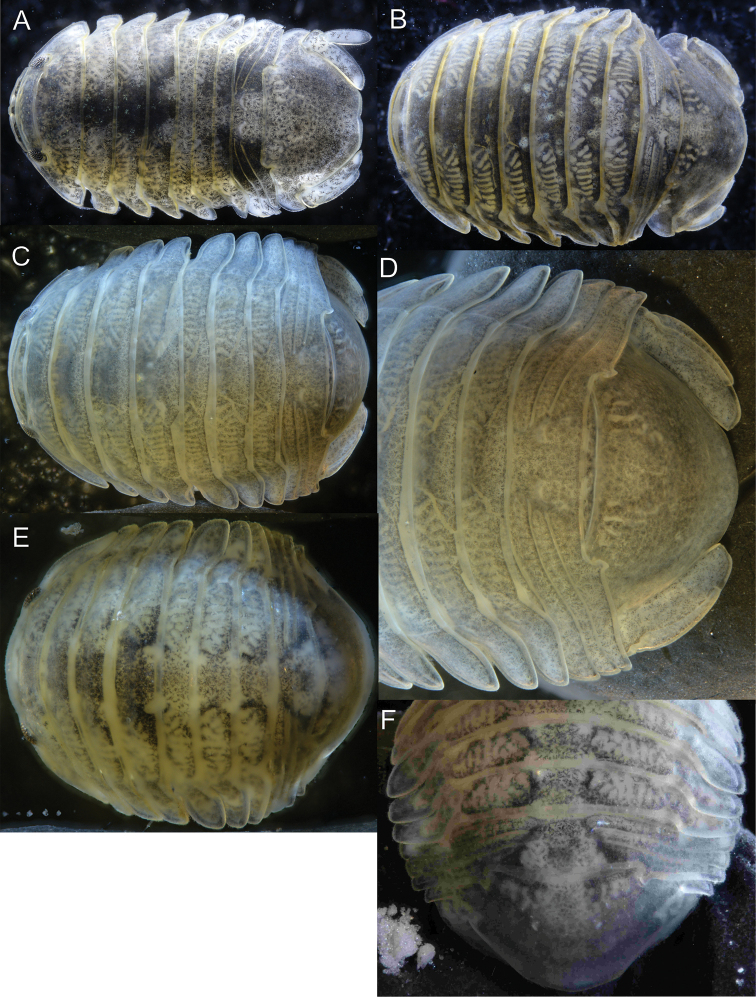
♂ *Gnorimosphaeroma* spp. Dorsal **A***Gnorimosphaeroma noblei*LACM:DISCO:220 **B***Gnorimosphaeroma oregonense*LACM:DISCO:11161 **C***Gnorimosphaeroma rayi*LACM:DISCO:2707 anterior end **D***Gnorimosphaeroma rayi* posterior LACM:DISCO:2707 **E**LACM:DISCO:232 *Gnorimosphaeroma* sp. anterior **F**LACM:DISCO:232 *Gnorimosphaeroma* sp. posterior.

**Figure 13.**
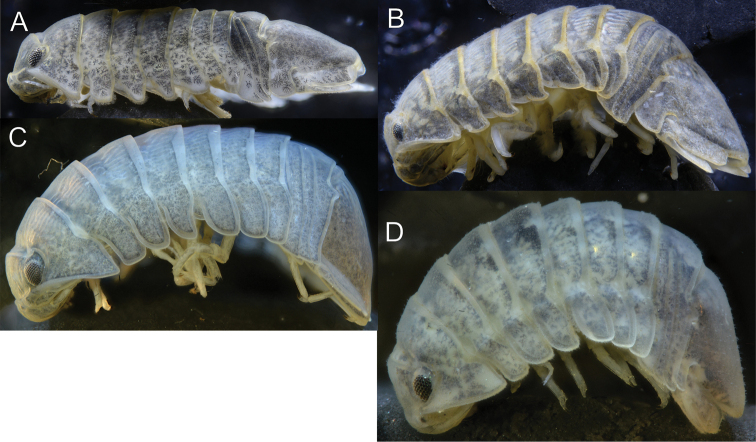
♂ *Gnorimosphaeroma* spp. Lateral view **A***Gnorimosphaeroma noblei*LACM:DISCO:220 **B***Gnorimosphaeroma oregonense*LACM:DISCO:11161 **C***Gnorimosphaeroma rayi*LACM:DISCO:2707 **D**LACM:DISCO:232 *Gnorimosphaeroma* sp.

Their very similar appearance to *G. oregonense* makes morphological identifications ambiguous, yet genetically they are easy to distinguish from *G. oregonense* (Figs 11–13, 15). Sequence divergence between the two species for the 16SrDNA fragment sequenced here is 16.5–20.9%. *Gnorimosphaeroma noblei* is always the sister taxon to *G. oregonense* in all of our genetic analyses (Fig. 11; Wetzer et al. 2018).

#### *Gnorimosphaeroma rayi* Hoestlandt, 1969

*Gnorimosphaeroma rayi* arrived in Tomales Bay in 1928 with oysters (*Crassostrea gigas* now accepted as *Magallana gigas* (Thunberg, 1793) from Japan (Bonnot 1935; Barrett 1963; James Carlton pers. comm. 2019). The type locality for this species is California, Marin County, Tomales Bay, Shallow Beach, 38.14°N, 122.881°W (Hoestlandt 1969). In addition to Japan, Hoestlandt (1975, 1977) reported this species from eastern Siberia and Hawaii. Hoestlandt too acknowledged the differences between *G. rayi* and *G. oregonense* are subtle. Hoestlandt’s (1975) key attempts to disambiguate the four species (*G. oregonense*, *G. noblei*, *G. rayi*, and *G. insulare*). However, we urge caution as his key may only be applicable to the largest specimens of each species, and we were unable to use it consistently.

Based on all of the material in the LACM collections available for genetic analysis, we could only confirm that the species occurs in Tomales Bay (three lots) and one lot from Bolinas Beach. Bolinas Beach is just 43.5 km south of Tomales Bay (Fig. 11, Table 1). It does not appear that this species is broadly distributed or quickly expanding its range (Figs 11–13, 15). A further assessment of its distribution awaits future genetically appropriately collected material and analyses. Additionally, we recognized a previously unidentified *Gnorimosphaeroma* sp. in San Francisco Bay. Genetically *Gnorimosphaeroma* sp. and *G. rayi* are readily distinguished and are always sister taxa in our analyses. However, we were unable to identify any reliable morphological characters to distinguish the two species. Based on the phylogenetic relationship between *Gnorimosphaeroma* sp. to *G. rayi*, it is presumed it too has a western Pacific origin.

#### *Gnorimosphaeroma* sp.

Morphologically this species cannot be distinguished from *G. rayi*. However, it is clearly genetically distinct with 13.9–16.5% sequence divergence for the 16S-rDNA fragment that was sequenced. Since we know *G. rayi* is an introduction from the western Pacific, this species is also likely a trans-Pacific traveler. San Francisco Bay, a biodiversity hotspot, is infamous for non-native and invasive species. At this time, there are no sequences available for western Pacific *Gnorimosphaeroma* that would allow identification of this species and clarification of their relationships (Figs 11–13). Many western Pacific species are poorly described and in need of redescription, making it impossible at this time to identify these specimens further.

#### *Gnorimosphaeroma insulare* (Van Name, 1940)

**Figure 14.**
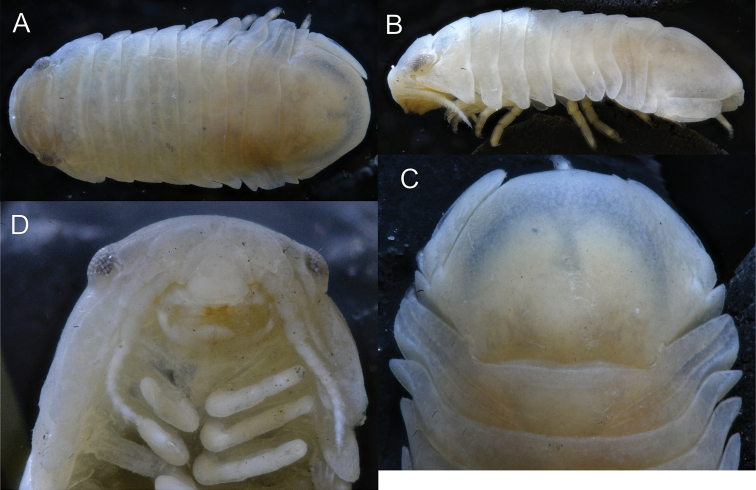
*Gnorimosphaeroma insulare*. Paratype. Male LACM:DISCO:6963 **A** dorsal **B** lateral **C** clypeus and labrum **D** pleotelson dorsal.

*Gnorimosphaeroma insulare* was described from freshwater on San Nicolas Island. San Nicolas is part of the Channel Island Archipelago off the Southern California Coast, today located nearly 100 km from the nearest point on the mainland coast. Menzies’ (1954) redescription detailed the *G. oregonense* distribution and compared *G. oregonense* to Van Name’s (1940) *Exosphaeroma insulare*, which Menzies moved to *Gnorimosphaeroma*. Menzies (1954) noted that the largest *G. insulare* is 8 mm in length. Some confusion then ensues with the description of *G. noblei* Menzies, 1954. *Gnorimosphaeroma noblei* is described from Tomales Bay from the high intertidal found in association with *Armadilloniscus*, hence associated with possible freshwater input and thus lower salinity. To the best of our knowledge, the only material of *G. insulare* is that from the original collections and type locality on San Nicolas Island. Eleven specimens were collected from freshwater where they were associated with the freshwater gastropod pulmonate, *Physa virgata* (Gould, 1855). The LACM collections hold a single male syntype which is photographed here (Fig. 14). Additional specimens are at the American Museum of Natural History. The specific collecting locality on San Nicolas is not known and it is unclear if any freshwater still runs today. Accessing this US Navy-controlled island, which is used for weapons testing and training, is difficult.

### Key

**Table:** 

1	Body length 2 × width. Body widest at pereonite 7 and anterior portion of pleon (Fig. 14A). Known only from a freshwater pond on San Nicolas Island	***Gnorimosphaeroma insulare***
–	Body more than 2 × width. Body widest at pereonite 6 or pereonites 2–7 similar in width	**2**
2	In lateral view, pereonite coxal plates 2, 3, and 4 anterior margins raised, posterior margin not raised, giving coxae a somewhat “s-shaped” appearance (Fig. 15A, C). Species is fully marine	***Gnorimosphaeroma oregonense***
–	In lateral view, pereonite coxal plates 2, 3, and 4 anterior margins not raised. Species may occur in marine, brackish, or freshwater	**3**
3	Pereonites 1–4 coxal plates margins with setose fringe (Fig. 15B). Posterior pleotelson margin with slight indentation (Fig. 12A). Species occurs in brackish or freshwater	***Gnorimosphaeroma noblei***
–	Pereonites 1–4 without setose fringe on coxal plate margins. Posterior pleotelson margin without indentation (Fig. 12D, F). Species are fully marine.	**4**
4	Pleonites lateral margins acute. Pleon lateral anterior margin smooth, without ornamentation (Fig. 12D)	***Gnorimosphaeroma rayi***
–	Pleonites lateral margins rounded. Pleonal lateral anterior margin with short acute lobe (Fig. 12F)	***Gnorimosphaeroma* sp.**

## Discussion

The genus *Gnorimosphaeroma* is a temperate-water clade occurring only on the shores of the northwestern Pacific (China, Japan), east through Alaska, and along the East Pacific coast to southern California shores. The genus is most speciose in the north western Pacific with 26 described species. Many of these species descriptions are inadequate, in need of critical evaluation, and redescription. In the eastern Pacific, *G. oregonense* is the most wide-ranging species, apparently occurring from Alaska to San Francisco, California. However, in this study we were only able to verify morphologically and genetically the species occurrences from Vancouver to San Francisco Bay. Adult specimens of *G. oregonense* become larger and more robust with increasing latitude. Along the Washington to California coast this species commonly co-occurs with *Exosphaeroma inornata* Dow, 1958. The latter is known from Puget Sound, Washington to central-southern Baja California Norte, Mexico (Wall et al. 2015). When specimens are very small and/or subadults, not only do the species of *Gnorimosphaeroma* get readily confused, but sometimes they are misidentified as *E. inornata* if the most careful attention is not paid. Any distinctive color patterns are lost in preserved material.

However, we have demonstrated that regardless of their size, species of *Gnorimosphaeroma* and *Exosphaeroma inornata* are readily distinguished based on their genetics (Wetzer et al. 2018). For both molecular analyses and morphological study, we had a very restricted distribution of *G. oregonense* specimens available. Kussakin 1979 had similarly struggled with resolving the identity of Northern Pacific *Gnorimosphaeroma*. He recognized that the genus contains fully freshwater, brackish, and marine species. *Gnorimosphaeroma kurilense* (Gurjanova, 1933) occurs in freshwater. It would be informative to be able to compare genetic sequences of our material with specimens from north of Vancouver, British Columbia to Alaska, fully marine to freshwater, and verify that the specimens observed and figured by Kussakin and others are genetically similar. Kussakin’s specimen(s) were from Popov Island, Primorsky Krai, near Vladivostok northwestern Pacific. The dorsal view (fig. 260, pg. 407) does look very similar to the *G. oregonense* from the eastern Pacific. It possesses the same distinctive rather heavily calcified coxal plates with strong carinae on coxae 2, 3, 5, and 6. Coxa 1 is acute, coxa 2 is subquadrate, with coxae 3–7 becoming more acute posteriorly. Kussakin’s figures (fig. 262) differ from our specimens in that antenna 1 has 15 flagellar articles and antenna 2 has ten flagellar articles compared to most of the specimens which we observed, which have 13 and 14, respectively. Also, the mandible of the Kussakin specimen has three long setae at the base of the molar incisor. Our specimens lack such setae (Fig. 4C, D). Pereopod 1 of the Popov specimen (fig. 261) appears more setose than the Washington specimen (Fig. 5A).

Since we only had specimens of *G. oregonense* available from a restricted range of Vancouver to Washington, we cannot assess the genetic diversity across the species’ larger range (Figs 10, 11). Our *Exosphaeroma amplicauda* (Wall et al. 2015) review resulted in the recognition of five species with Alaska and Washington specimens recognized as distinct from the type locality (Central California), and distinct from those from the Southern California coast. It would not be surprising if future studies based on broader sampling revealed greater genetic diversity than we have observed here.

We also had available for study a single male syntype of *G. insulare* Van Name, 1940 (Fig. 14). As noted previously by Van Name, *G. insulare* and *G. oregonense* are very similar. Examination of the specimens we had available, dorsally *G. insulare* appears oblong and is ca. twice as long as wide compared to *G. oregonense*, *G. noblei*, *G. rayi*, and the unidentified *Gnorimosphaeroma* sp. from San Francisco Bay. These species are all broader than *G. insulare* and therefore have a more globular appearance. The largest *G. insulare* specimen observed was 8 mm in length, whereas the largest known individuals of *G. oregonense*, *G. noblei*, and *G. rayi* had been previously recorded as 12 mm in length. *Gnorimosphaeroma insulare* is distinguished from all other *Gnorimosphaeroma* species in that it appears to have been entirely restricted to a freshwater pond and only known from the type locality. It is unknown whether this pond still exists today. Since it is the sole specimen (syntype) and fragile, no dissections were undertaken, but rather the specimen was photographed (Fig. 14). A collection made on San Miguel Island by E. Hochberg and identified by E.W. Iverson was reidentified here as *G. noblei*. Sadly, no habitat information was provided for this collection and it had been formalin fixed and is not available for genetic study. San Miguel Island is the northernmost of the Channel Islands and 74 miles distant from San Nicolas Island. The only specimens known from offshore islands were these two lots.

Future genetic comparisons of marine, brackish, and freshwater *Gnorimosphaeroma* species occurring north of Vancouver, through Alaska to Primorsky Krai (northwest Pacific) may reveal either multiple invasions or a single invasion to brackish and freshwater and may change the current phylogenetic relationship of brackish/freshwater species and marine species in the Eastern Pacific. Phylogenetic placement of *G. insulare* would also be most interesting should populations at this locality still exist today.

**Figure 15.**
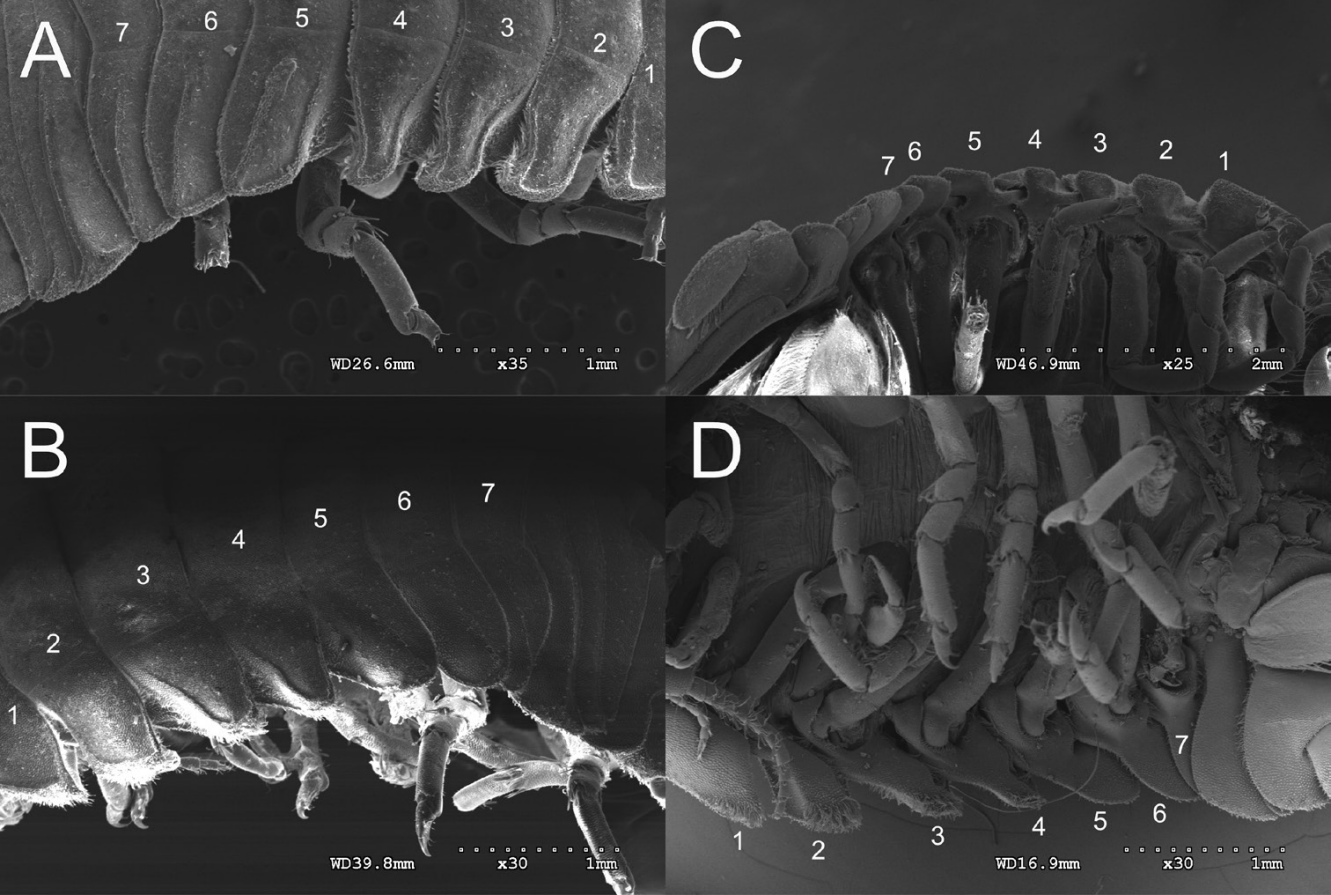
SEM comparison of *G. oregonense*LACM:DISCO:11164 and *G. noblei*LACM:DISCO:11168 coxae **A***G. oregonense* lateral **B***G. noblei* lateral **C***G. oregonense* ventral, and **D***G. noblei* ventral.

Identification keys for west coast *Gnorimosphaeroma* species are available in Menzies (1954) and Hoestlandt (1975, 1977). Kussakin (1979) provides a key for north Pacific species, Kwon and Kim (1987) for Korean species, and Nunomura (1998) for the Japanese species. Difficulty arises in using them as differences between species can be very subtle and may only apply to very large adult specimens. In some instances, the largest specimens possible for the species may not have been available at the time of description (e.g., *G. noblei* Hoeslandt, 1975). However, the differences in the lateral and ventral appearance of the coxal plates of *G. oregonense* and *G. noblei* are distinct in large adult males and females (Figs 13A, B, 15). In *G. oregonense* lateral view the anterior margins of coxal plates 2, 3, and 4 are raised, posterior margin not raised, giving coxae a somewhat “s-shaped” appearance (Fig. 15A, C). Ventrally these appear as interlocking units. *Gnorimosphaeroma noblei* which can co-occur with *G. oregonense* lacks these (ventrally coxae not interlocking) (Fig. 15B, D). The two species are also readily distinguished based on habitat and salinity. *Gnorimosphaeroma oregonense* is always in fully marine waters and *G. noblei* inhabits high intertidal, brackish to fully freshwaters.

As molecular phylogenetic studies allow more and deeper sampling, cryptic species in marine environments are being recognized with ever greater frequency. Organisms as diverse as foraminiferans (Aurahs et al. 2009), copepods (Bláha et al. 2010), hydroids (Moura et al. 2008), and valviferan isopods (Xavier et al. 2012) are revealing much greater diversity than previously recognized. This diversity is and cannot always be recognized morphologically. The recent detailed study of the sphaeromatid isopod *Dynamene* by Vieira et al. (2019) demonstrated that not only can large sequence divergences exist over small spatial scales, but that repeated invasions leave their genetic mark on populations, and that population diversification can be recognized over shorter time scales than previously thought for organisms with limited dispersal abilities. Based on the putative cryptic species in their study, they estimate a 300% under-estimation of known species in *Dynamene*, a species-poor genus. As more *Gnorimosphaeroma* species and specimens for genetic analysis become available, this genus has the potential to provide interesting insights into not only the evolution of the rare marine to freshwater invasion of species within the genus, but also human induced species relocations across the Pacific Ocean. If *G. insulare* still exists on San Nicolas Island and possibly on other Channel Islands too, this genus could reveal a very interesting phylo-biogeographic history.

## Supplementary Material

XML Treatment for Gnorimosphaeroma

XML Treatment for Gnorimosphaeroma oregonense
